# Dipeptidyl peptidase-4 plays a pathogenic role in BSA-induced kidney injury in diabetic mice

**DOI:** 10.1038/s41598-019-43730-5

**Published:** 2019-05-17

**Authors:** Yuta Takagaki, Sen Shi, Makoto Katoh, Munehiro Kitada, Keizo Kanasaki, Daisuke Koya

**Affiliations:** 1 Department of Diabetology and Endocrinology, Kanazawa Medical University, Uchinada, Ishikawa 920-0293 Japan; 2 Division of Anticipatory Molecular Food Science and Technology, Kanazawa Medical University, Uchinada, Ishikawa 920-0293 Japan; 3 Mitsubishi Tanabe Pharma Corporation Ikuyaku, Integrated Value Development Division, Tokyo, Japan

**Keywords:** End-stage renal disease, Renal fibrosis

## Abstract

Diabetic kidney disease (DKD) is appeared to be higher risk of declining kidney function compared to non-diabetic kidney disease with same magnitude of albuminuria. Epithelial-mesenchymal transition (EMT) program of tubular epithelial cells (TECs) could be important for the production of the extracellular matrix in the kidney. Caveolin-1 (CAV1), dipeptidyl peptidase-4 (DPP-4) and integrin β1 have shown to be involved in EMT program. Here, we found diabetic kidney is prone for albuminuria-induced TECs damage and DPP-4 plays a vital role in such parenchymal damages in diabetic mice. The bovine serum albumin (BSA) injection induced severe TECs damage and altered expression levels of DPP-4, integrin β1, CAV1, and EMT programs including relevant microRNAs in type 1 diabetic CD-1 mice when compared to non-diabetic mice; teneligliptin (TENE) ameliorated these alterations. TENE suppressed the close proximity among DPP-4, integrin β1 and CAV1 in a culture of HK-2 cells. These findings suggest that DPP-4 inhibition can be relevant for combating proteinuric DKD by targeting the EMT program induced by the crosstalk among DPP-4, integrin β1 and CAV1.

## Introduction

Albuminuria is the risk factor for kidney parenchymal damage^1^. Among the same magnitude of albuminuria stage, patients with diabetic kidney disease (DKD) appear to be higher risk in the progression of declining in kidney function when compared to non-diabetic kidney disease patients^2^. However clear molecular mechanisms why diabetes is prone for the renal functional decline are not completely elucidated yet.

Kidney fibrosis is a characteristic feature of advanced kidney diseases, such as DKD^3^. Although fibrosis is essentially a tissue repair process, progressive kidney fibrosis may be a consequence of a disruption in the normal wound-healing process^4,5^. The phenotype of damaged kidney cells changes into a matrix-producing mesenchymal-like phenotype of the so-called myofibroblast^3^. The presence of epithelial-mesenchymal transition (EMT)-derived fibroblasts remains controversial^6^. However, the numbers of renal tubular epithelial cells (TECs) with EMT features have shown to be associated with the serum creatinine level and the degree of interstitial damage^7^. Thus, EMT programs, even partial programs, may be relevant for the pathogenesis of renal fibrosis. Snail and ZEB1 are zinc-finger transcription factors that are known to bind to the promoter sequence of E-cadherin and induce the EMT program. Twist1 is a bHLH transcription factor that is known to suppress the expression of E-cadherin and induce the EMT program^8^. In mice, a conditional deletion of Twist1 or Snai1 in proximal TECs results in the inhibition of the EMT program and renoprotection^7^. Therefore, Snail, ZEB1 and Twist1 are relevant for the pathogenesis of kidney damage associated with EMT program. MicroRNA-200 and miR-34 have been shown to regulate the TGF-β-induced EMT program by suppressing the transcription factors ZEB1 and snail^9^. TECs undergoing EMT are associated with an overall loss of aquaporin 1 (AQP1)^10^, which influences cell proliferation and cell cycle progression^11^. Transforming growth factor (TGF)-β induces the EMT program and attenuates the self-repairing potential of the epithelial cell^10^.

Dipeptidyl peptidase (DPP)-4, which was originally characterized as a T-cell differentiation antigen (CD26), is also a membrane-binding protein that interacts with intra- and extracellular molecules. In addition to degrade incretin hormones, interestingly, DPP-4 exerts diverse effects via both its enzymatic activities and non-enzymatic actions, such as binding to other binding partners, including other membrane-binding molecules and extracellular molecules^12^. In the mammalian kidney, the expression of DPP-4 is the highest per organ weight and is increased in type 1 and type 2 diabetic models^13–15^. Our group previously reported that the DPP-4 inhibitor linagliptin suppresses the expression of DPP-4 in the kidney by inducing miR-29 and restoring streptozotocin (STZ)-induced kidney fibrosis in CD-1 mice associated with the inhibition of the endothelial-mesenchymal transition (EndMT) and TGF-β/smad3 signaling^15^. Furthermore, the interaction between DPP-4 and integrin β1 regulates the key endothelial cell signal transduction inducing EndMT^16^.

Caveolin-1 (CAV1) is a scaffolding protein within the caveolae plasma membranes. The caveolae are specialized lipid rafts known to be important for cholesterol metabolism and various cell signaling transduction pathways, such as insulin signaling pathways^17–19^. CAV1 is also known to act as a signaling protein that interacts with intra- and extra-cellular molecules. CAV1 binds to the DPP-4 serine catalytic site^20^. CAV1 has been shown to increase during EMT and influence cancer cell adhesion^21^. Additionally, CAV1 and integrin β1 can stimulate ES cell proliferation under high glucose conditions by modulating the focal adhesion signaling pathways^22^. Proteinuria is the significant insult to the kidney and is associated with chronic kidney disease (CKD), such as DN^23^.

Here, we hypothesize that diabetic kidney is preconditioning for proteinuria-induced parenchymal damage and DPP-4 play essential roles in such kidney damage program in diabetic kidney.

## Results

### The DPP-4 inhibitor teneligliptin suppressed the bovine serum albumin (BSA)-induced tubular damage and fibrosis

**Figure 1:**
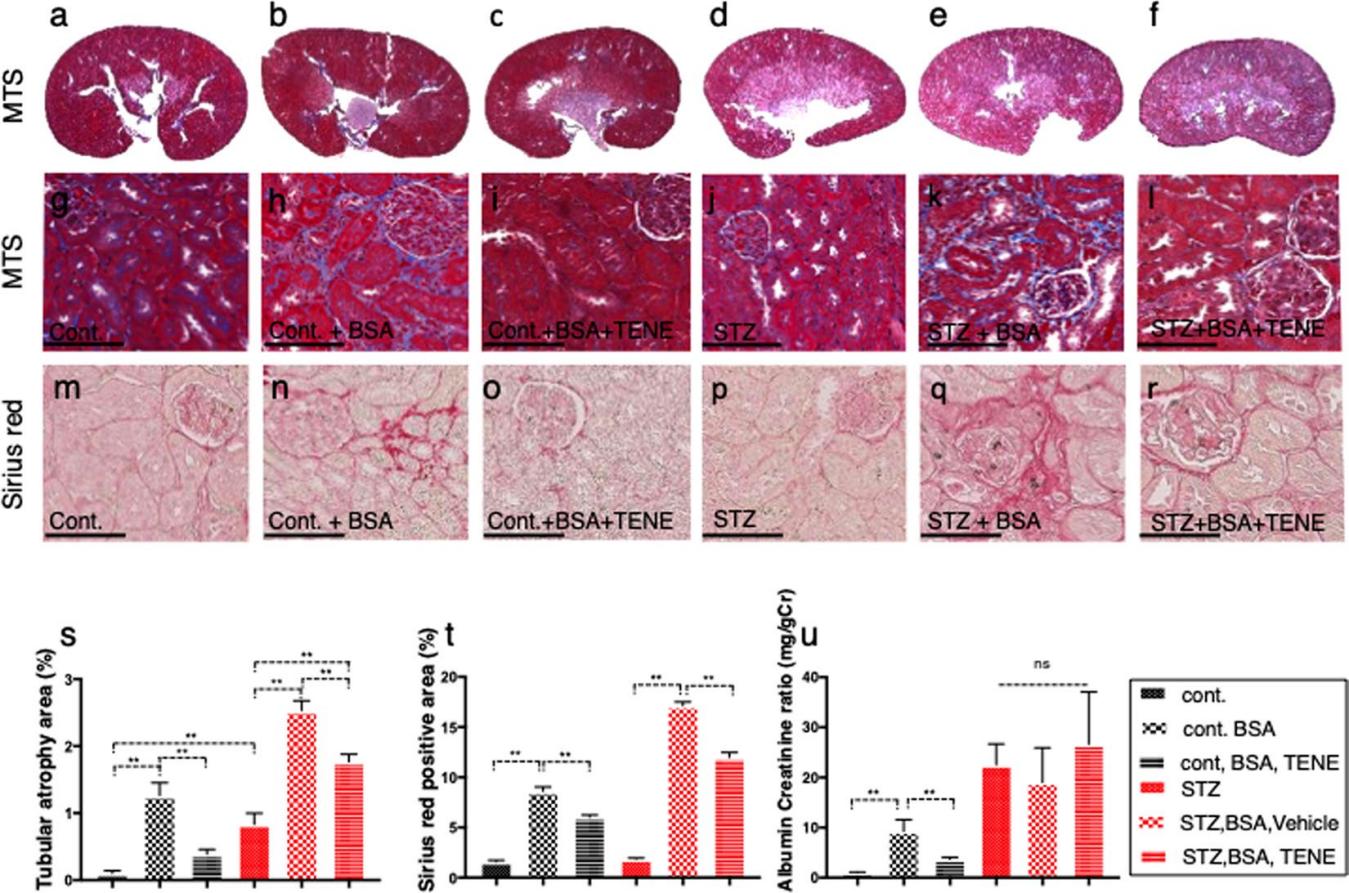
Diabetic mice exhibited accelerated BSA injection-induced proximal tubular damage and renal interstitial fibrosis; TENE treatment ameliorated these lesions. (**a**–**r**) Representative images (seven visual fields of each tissue were analyzed) of MTS (**a**–**l**) and SR staining (**m**–**r**) of kidneys from the indicated experimental groups. Scale bars, 100 μm. (**s**) Tubular atrophy area. Each group, *n* = 7. (0 = no tubular atrophy, 1 = tubular atrophy in up to 15% of the proximal tubules, 2 = tubular atrophy in 16–30% of the proximal tubules, and 3 = tubular atrophy in more than 30% of the proximal tubules). (**t**) SR staining positive area. (**u**) Urine murine albumin levels. Each group, *n* = 5. (**m**–**o**), unpaired two-tailed *t*-test. *P < 0.05, **P < 0.01. Data are presented as mean ± s.e.m.

**Figure 2:**
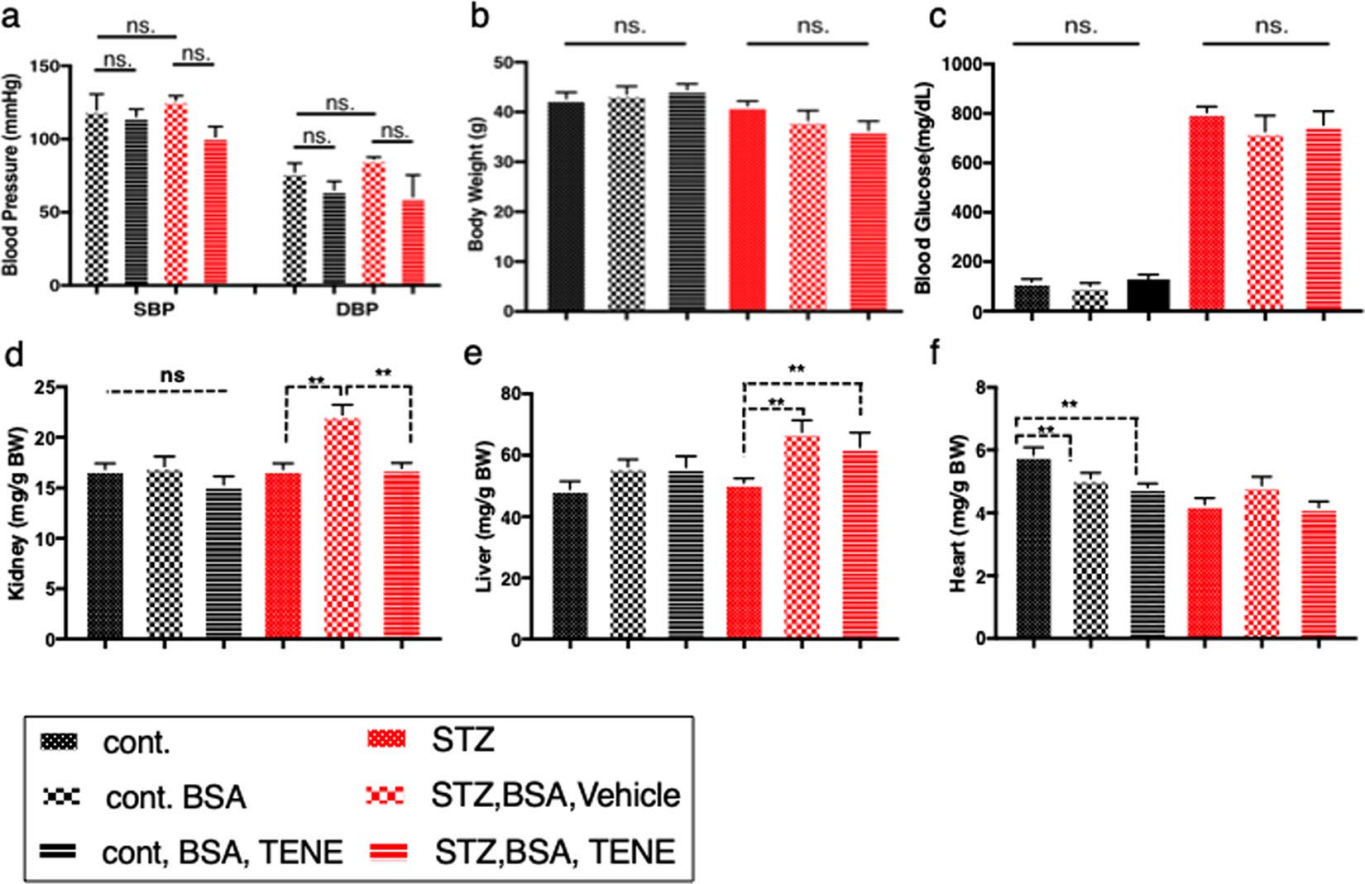
Characteristics of the mice in each experimental group. (**a**) Systolic blood pressure (SBP) and diastolic blood pressure (DBP) were measured 10 times, *n* = 3. (**b**) Body weight, (**c**) BS, (**d**) kidney weight (Bilateral), (**e**) liver and (**f**) heart weight are shown. Organ weights are shown as the organ to body weight ratio. Each group, *n* = 7, was analyzed with an unpaired two-tailed *t*-test. *P < 0.05, **P < 0.01. Data are presented as mean ± s.e.m.

At sacrifice, we obtained the following 6 groups: control, control + BSA, control + BSA + teneligliptin (TENE), STZ, STZ + BSA, and STZ + BSA + TENE. The histopathological examination of the kidneys using the Masson’s trichrome staining (MTS) and picrosirius red (SR) staining revealed that the BSA injection induced mild tubular atrophy (Fig. 1a–c,g–i,m–o,s) and interstitial fibrosis (Fig. 1g–i,m–o,t) in the control mice as previously reported^24^. The treatment with the DPP-4 inhibitor TENE ameliorated these alterations (Fig. 1c,i,o,s,t). In this short interval experiment, the STZ-induced diabetic mice that did not receive the BSA injection displayed a minor phenotype (Fig. 1d,j,p,s,t). Compared to the diabetic mice without BSA injection, the BSA-injected diabetic mice showed remarkable tubular damage and interstitial fibrosis; TENE treatment ameliorated these fibrogenic alterations (Fig. 1e,f,k,l,q,r,s,t). We also analyzed heart and liver histopathology; in this short duration of experimental protocol, there were minor differences between all groups analyzed in hearrt and liver (Supplementary Fig. 1). Compared to the control mice, the BSA-injected control mice displayed higher urine mouse-specific albumin excretion; TENE ameliorated the BSA-induced urine albumin levels (Fig. 1u). Although the diabetic mice displayed higher urine albumin excretion than the control mice, no significant difference was observed among all diabetic mice (Fig. 1u). No significant difference was observed in the urine BSA levels among all BSA-injected groups regardless of diabetes or TENE treatment (Supplementary Fig. 2). No significant difference was observed in the blood pressure, body weight (BW) and blood glucose (BG) between the BSA-injected control and diabetic mice (Fig. 2a–c). The kidney weight did not significantly differ among the control groups (Fig. 2d). In the STZ-induced diabetic mice, the BSA injection increased the kidney/BW ratio and liver weight/BW ratio. The TENE treatment decreased the kidney weight/BW ratio (Fig. 2d,e). Compared to the control mice, the heart weight was decreased trend in the diabetic groups (Fig. 2f). The biomarkers of liver and heart damage such as alanine aminotransferase (ALT), aspartate aminotransferase (AST) and N-terminal prohormone brain natriuretic peptide (NT-proBNP) were analyzed and found no significant differences between all groups (Supplementary Fig. 3).

### DPP-4 is involved in the mechanisms of the BSA-induced TGF-β/smad3 signaling pathway and EMT program of kidney fibrosis in diabetes

**Figure 3:**
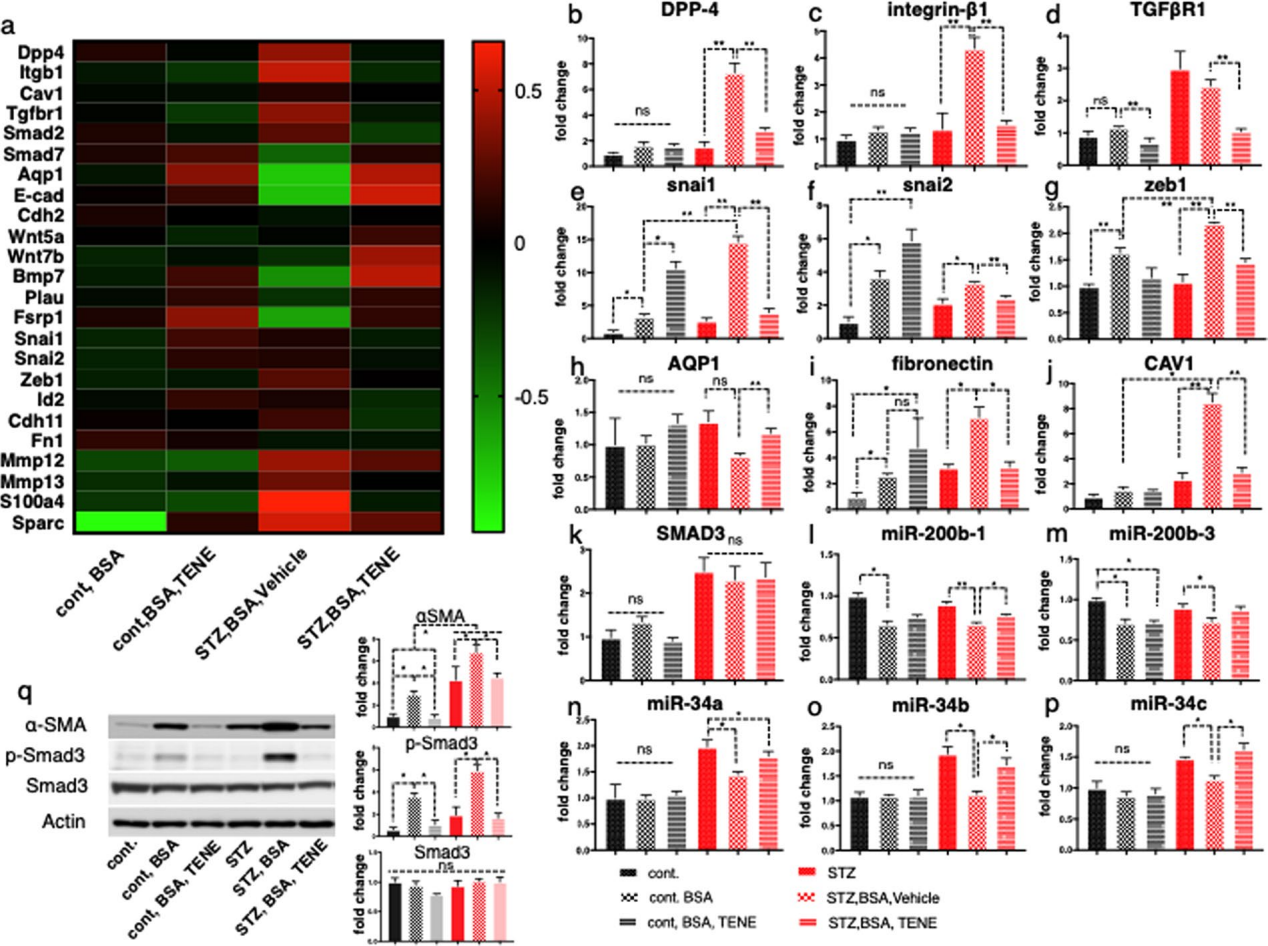
Fibrogenic gene and EMT-related gene expression levels were induced in the BSA-injected diabetic mouse kidney; TENE treatment suppressed these gene expression levels. (**a**) Microarray analysis of the kidney samples. Heat map analysis of the gene expression. BSA injection (particularly in the diabetic mice) induced genes, such as DPP-4, TGF-β/smad3 signaling, CAV1, integrin β1 and the EMT program; TENE treatment suppressed these alterations (*n* = 2 mice per group, and the average value is shown in the figure). Red indicates high and green indicates low relative expression levels. (**b**–**p**) qPCR analysis of the expression of the indicated genes in the kidney of mice in each group (*n* = 7 mice per group). Gene expression was normalized to the control mice value. *P < 0.05, **P < 0.01. Data are presented as mean ± s.e.m. (**q**) Representative western blot analysis. As a densitometric analysis, each protein level was normalized with actin. *n* = 7 per group were analyzed.

To explore the pathological role of DPP-4 in kidney fibrosis in the BSA-injected mice and the molecular mechanisms underlying the renoprotective effect of the DPP-4 inhibitor, we performed a microarray analysis and compared the gene expression profiles in the kidneys from BSA-injected diabetic and control mice (Fig. 3a). According to the microarray analysis, the BSA injection into the diabetic mice led to an induction of DPP-4, integrin β1, the TGF-β/smad3 signaling pathway and the EMT program (induction of EMT-related gene and mesenchymal markers and suppression of epithelial marker). qPCR analysis was performed, and compared to the BSA-injected control mice, the BSA-injected diabetic mice exhibited an induction of DPP-4, integrin β1 and TGF-β receptor 1 genes (Fig. 3b–d). These alterations were reversed by the TENE treatment. In the diabetic mice, the BSA injection increased the levels of EMT-associated genes, such as snai-1, snai-2, and Zeb-1, which encoded snail, slug and zeb-1. These changes were reversed by the TENE treatment (Fig. 3e–g). In contrast, the expression of the proximal tubular marker proteins AQP1 was decreased in the BSA-injected diabetic mice and increased by the TENE treatment (Fig. 3h). In the control mice BSA injection increased fibronectin levels; TENE did not suppress the level of fibronectin in BSA injected control mice. Diabetes alone did not increased fibronectin levels. BSA-injected diabetic mice exhibited elevated levels of fibronectin; TENE significantly suppressed fibronectin levels (Fig. 3i). Unexpectedly, the expression levels of the EMT markers snai-1 and snai-2 were increased in the BSA-injected control mice treated with TENE, whereas the mesenchymal markers, such as cadherin 11 and fibronectin 1, and the EMT marker ZEB-1 were unaltered (Fig. 3a,e–i). After analyzing the involvement of DPP-4 in the EMT program in the kidney, we became interested in CAV1, which is a scaffolding protein within the caveolae plasma membranes. The expression pattern of CAV1 was similar to that of DPP-4 and integrin β1 (increased by the BSA injection and diabetes and decreased by the TENE treatment) (Fig. 3a,j). The gene expression of smad3 was increased by diabetic group but not altered by either BSA injection or TENE treatment (Fig. 3k).

To analyze the molecular mechanism by which TENE suppressed the EMT program, we focused on the miR-34 and -200, the miRs relevant for EMT program^25^. Compared to the control mice, the BSA-injected diabetic mice exhibited lower expressions of the miR-34 and -200, while TENE restored these expressions (Fig. 3l–p). Therefore, TENE suppressed the EMT program induced by the BSA injection in the diabetic kidney via the induction of anti-EMT miRs. miR-29s, the anti-fibrotic miRs^26^, levels were displayed a parallel trend with tissue damage (Supplementary Fig. 4)^15,16,27^. Western blot analyze revealed that smad3 phosphorylation and αSMA protein expression were induced by the BSA injection in both the control and diabetic groups; TENE treatment reversed these changes (Fig. 3q).

**Figure 4:**
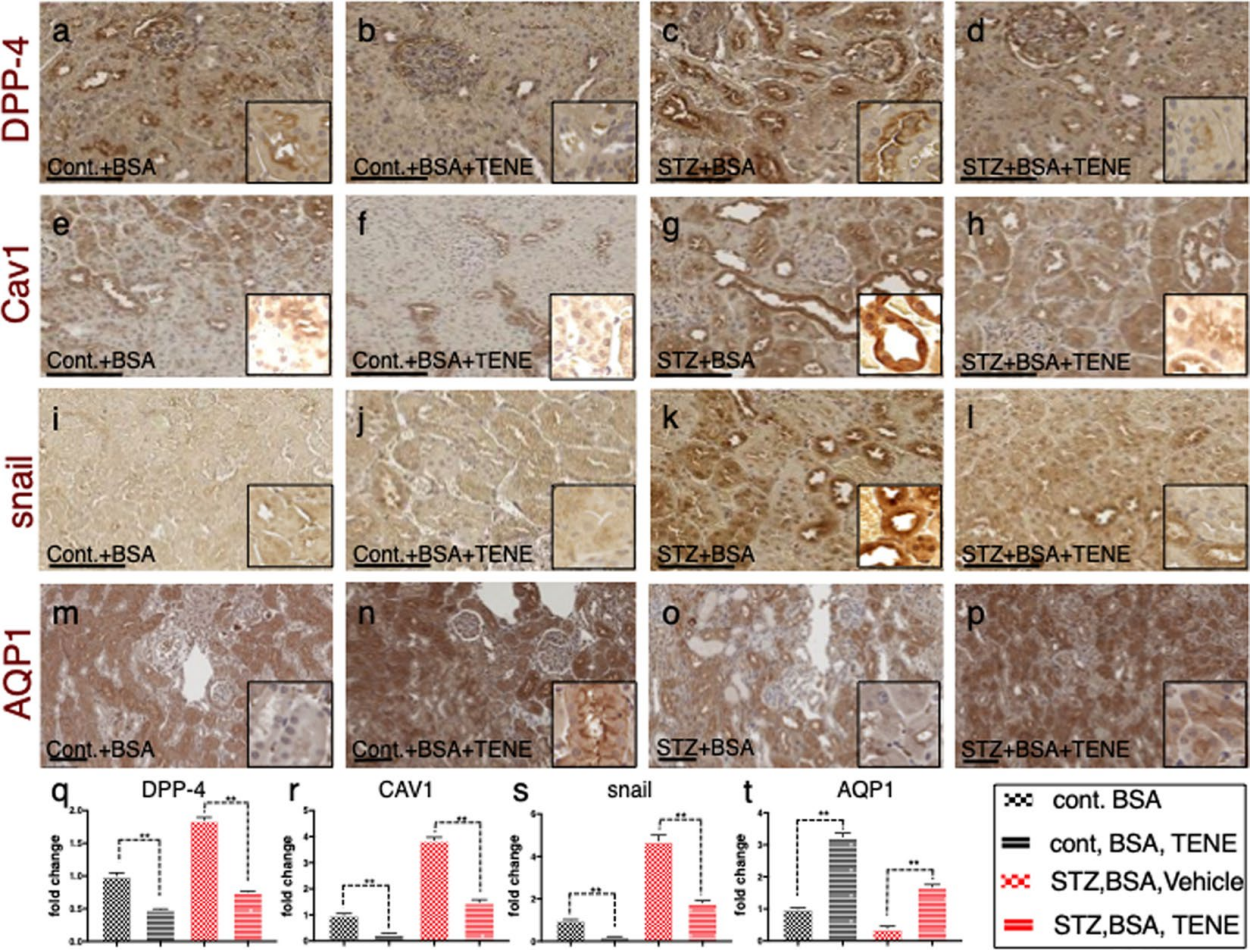
BSA-injected diabetic mice exhibited high tubular levels of DPP-4, CAV1 and EMT program; TENE treatment ameliorated these alterations. Immunohistochemical analysis of (**a**–**d**) DPP-4, (**e**–**h**) CAV1, (**i**–**l**) snail and (**m**–**p**) AQP-1 from the BSA-injected control or diabetic mice with or without the TENE treatment. Scale bar, 50 μm. Representative images from *n* = 7 in each group are shown. Each group was analyzed with an unpaired two-tailed *t*-test. *P < 0.05, **P < 0.01. Data are presented as mean ± s.e.m (**q**–**t**).

**Figure 5:**
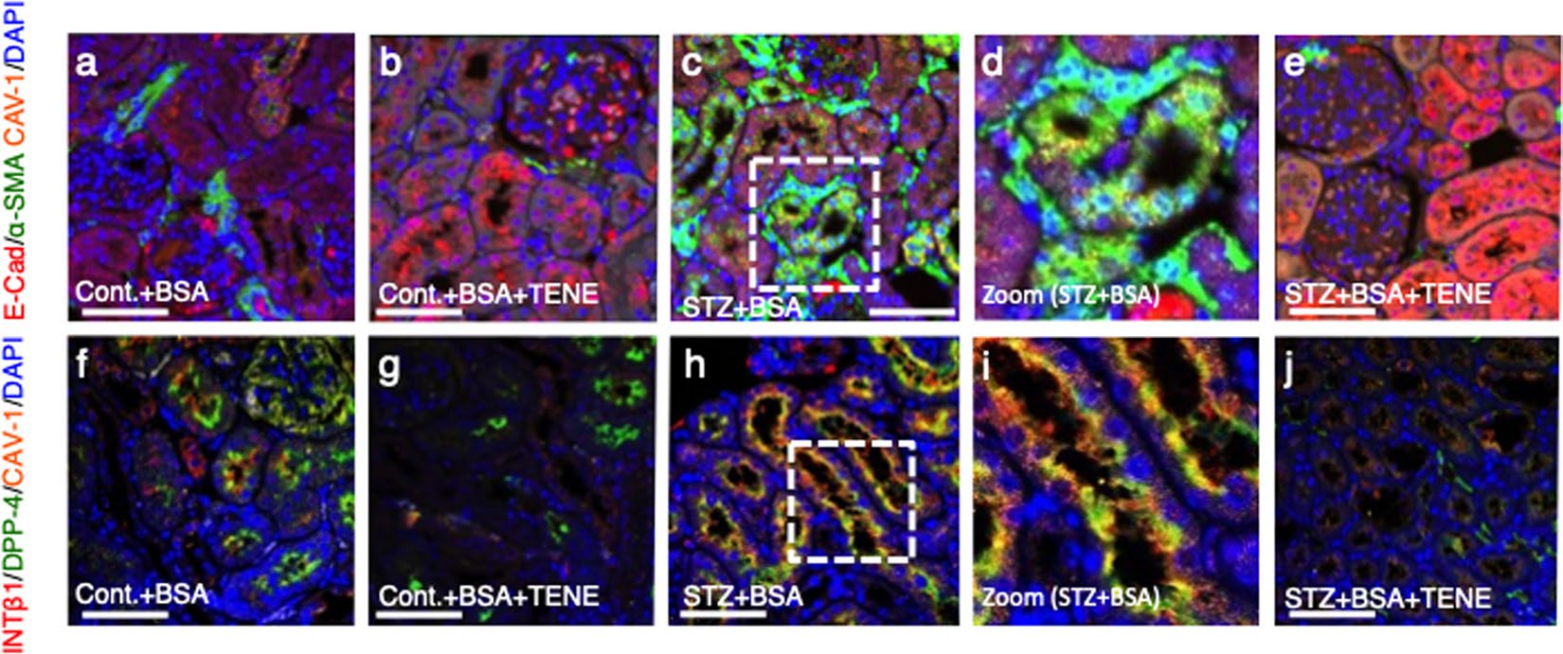
EMT in BSA-induced damaged tubule was associated with increased levels of DPP-4, integrin β1 and CAV1; TENE treatment ameliorated these alterations. (**a**–**e**) Multiplex immunofluorescence microscopy analysis of the EMT program and association with CAV1. Formaldehyde-fixed, paraffin-embedded (FFPE) kidney samples were labeled with epithelial markers for E-cadherin, αSMA and CAV1. An immunofluorescence analysis was performed by confocal microscopy. (**d**) The enlarged image of the inset shown in (**c**). The αSMA-positive damaged tubular cells were surrounded by αSMA-positive interstitial cells (**f**–**j**). Multiplex immunofluorescence was performed to analyze the crosstalk among DPP-4, integrin β1 and CAV1 in the BSA-injected diabetic mice. (**i**) The enlarged image of the inset shown in (h). DPP-4, integrin β1, and CAV1 were localized at the same location (likely the luminal side of the proximal tubule). The crosstalk occurred more frequently in the damaged tubular cells. Representative images from *n* = 7 in each group are shown.

Similar to the observations in the microarray analysis, the immunohistochemistry analysis revealed that the DPP-4 and CAV1 expression levels were higher in the tubular epithelium in the BSA-injected diabetic mice than those in the BSA-injected control mice (Fig. 4a,c,e,g,q,r, Supplementary Fig. 5). Compared to the BSA-injected control mice, the BSA-injected diabetic mice exhibited an induction of snail and suppression of AQP1 levels (Fig. 4i,k,m,o,s,t, Supplementary Fig. 5); TENE treatment restored these alterations (Fig. 4a–t, Supplementary Fig. 5). These alterations of protein levels of DPP-4 and CAV1 in each group were confirmed by the western blot analysis (Supplementary Fig. 6). The multiplex staining with E-cadherin, αSMA and CAV1 revealed that compared to the BSA-injected control mice, the BSA-injected diabetic mice displayed E-cadherin, αSMA and CAV1 triple-positive cells in the kidney, suggesting that the EMT program is associated with the induction of CAV1. The TENE treatment inhibited the EMT programs and CAV1 levels (Fig. 5a–e). Furthermore, the damaged tubules exhibited a higher expression of DPP-4, integrin β1 and CAV1 (Fig. 5f,h–i), while the TENE treatment ameliorated these alterations (Fig. 5g,j). Thus, the EMT program induced in the damaged kidney tubular cells was associated with the crosstalk among DPP-4, integrin β1 and CAV1, and the TENE treatment suppressed the EMT program by inhibiting this crosstalk.

### TGF-β treatment induced DPP-4-dependent interaction among DPP-4, integrin β1 and CAV1 in the epithelial cells

**Figure 6:**
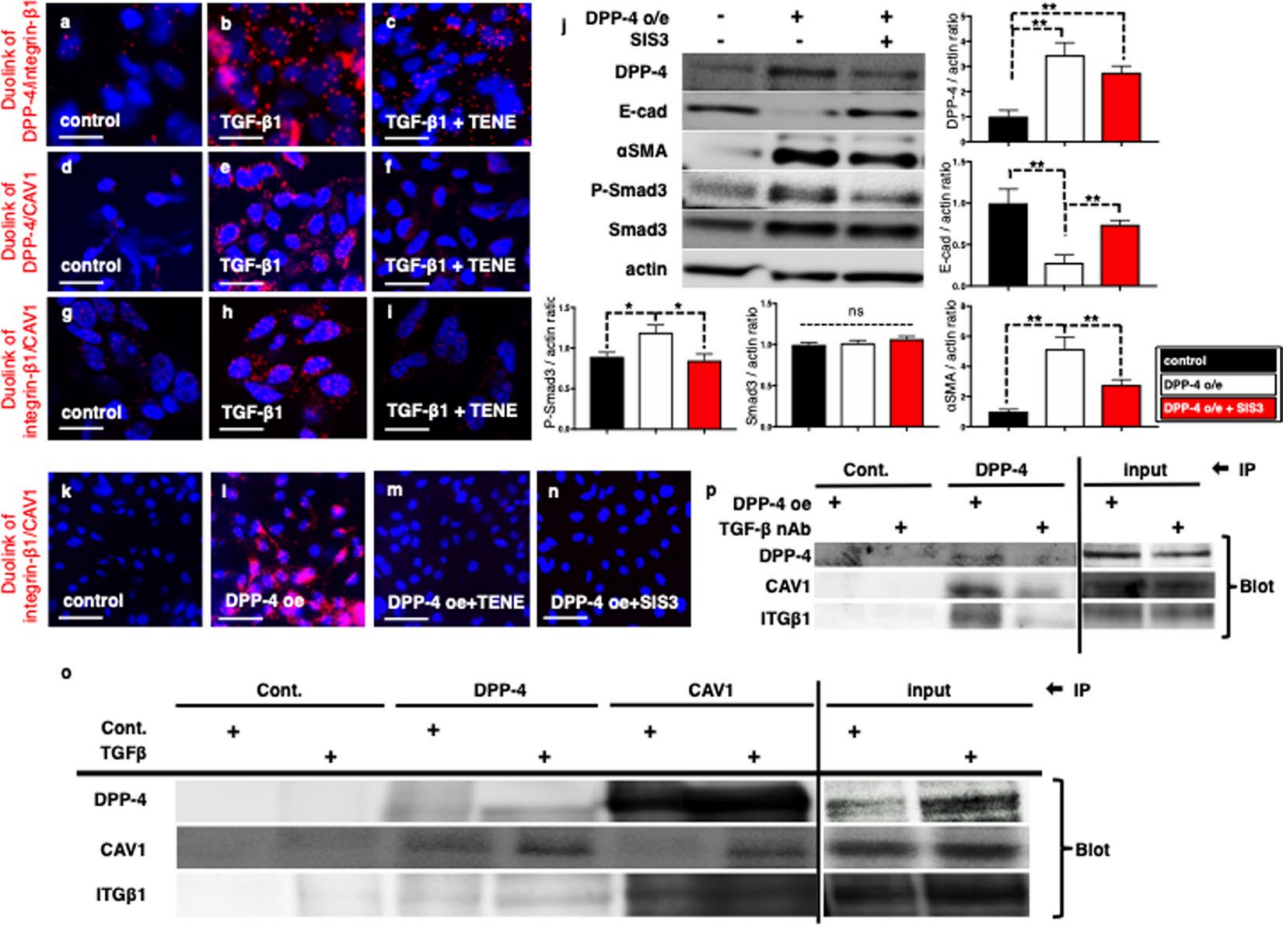
TENE treatment suppressed the crosstalk among DPP-4, integrin β1 and CAV1 via inhibition of TGF-β/smad3 signaling pathway *in vitro*. Duolink *in situ* analysis of (a-c) DPP-4/integrin β1, (**d**–**f**) DPP-4/CAV1 and (**g**–**i**) integrin β1/CAV1 in HK-2 cells with or without TGF-β1 (10 ng/ml) was performed by confocal microscopy (×1260). Scale bar: 50 μm in each panel. (**j**) Representative western blot analysis. As a densitometric analysis, each protein level was normalized with actin. n = 6 per group were analyzed. (**k**–**n**) Duolink *in situ* analysis of integrin β1/CAV1 in DPP-4 overexpressed HK-2 cells with or without TENE and SIS3. (**o**) Immunoprecipitation analysis revealed TGF-β treatment increased crosstalk among DPP-4, integrin β1 (ITGβ1) and CAV1. (**p**) Immunoprecipitation assay revealed TGF-β neutralization suppressed crosstalk among DPP-4, integrin β1 and CAV1 induced by DPP-4 overexpression.

To confirm the crosstalk among DPP-4, integrin β1 and CAV1, a Duolink *In Situ* proximity ligation assay was performed. Similar to our previous report in endothelial cells, TGF-β1 induced close proximity between DPP-4 and integrin β1, while TENE suppressed the TGF-β1-induced proximity in HK2 cells (Fig. 6a–c). Furthermore, we found that CAV1 and either DPP-4 or integrin β1 displayed close proximity as a result of the TGF-β1 stimulation, while TENE inhibited the proximities of these molecules (Fig.6d–i). The overexpression of CAV1 induced close proximity between DPP-4 and integrin β1; DPP-4 overexpression induced close proximity between integrin β1 and CAV1, while TENE suppressed them (Supplementary Fig. 7). In HK-2 cell, DPP-4 overexpression decreased E-cadherin, increased αSMA (the induction of EMT) and increased Smad3 phosphorylation; SIS3, the selective inhibitor of TGF-β1 dependent smad3 phosphorylation, suppressed EMT program (Fig. 6j). DPP-4 overexpression-induced close proximity between integrin β1 and CAV1 was suppressed with SIS3 (Fig. 6k–n). Immunoprecipitation assay further revealed that TGF-β stimulation induced physical interaction among DPP-4, CAV1 and integrin β1 (Fig. 6o). Finally we confirmed that neutralization of TGF-β decreased the physical interaction between DPP-4, integrin β1 and CAV1 induced by DPP-4 overexpression (Fig. 6p), supporting the significance of TGF-β/smad3 signaling pathway in the crosstalk among these three molecules.

## Discussion

Diabetic patients with macroalbuminuria have a poor kidney prognosis^28–30^. Therefore, establishing a novel therapeutic strategy for diabetic patients with advanced albuminuria or proteinuria appears to be highly significant in diabetic research. Our research group has focused on the endothelium and reported that DPP-4 plays fibrogenic roles by inducing EndMT, which is associated with the suppression of anti-fibrogenic miR crosstalk^31–33^. Furthermore, we reported that the interaction between DPP-4 and integrin β1 regulates TGF-β/smad3 signal transduction and induces EndMT^16^. In this study, we focused on the proximal tubular epithelium where the cells are exposed to diverse urine derived molecules, including albumin. We found that (1) Diabetic mice exhibited severe fibrosis by BSA injection when compared to BSA injected control mice associated with induction of EMT program, (2) the TENE treatment ameliorated the proximal tubular damage and tubulointerstitial fibrosis induced by the BSA injection in the control and diabetic mice, (3) the TENE treatment suppressed the EMT program induced by the BSA injection in the diabetic mice by increasing anti-EMT miRs and (4) The crosstalk among DPP-4, integrin β1 and CAV1 was TGF-β/smad3 signaling dependent. These data provide novel insights into the pathogenesis of DKD and the pathogenic role of DPP-4 in the progression of DKD.

In our study, the BSA-stimulated fibrogenic/EMT molecular inductions were rather prominent in the STZ-induced diabetic mice. This phenomenon is clinically relevant since DKD with albuminuria is an independent risk factor for eGFR decline compared to non-diabetic CKDs with similar levels of albuminuria^2,29^. Furthermore trends of higher risk in the onset of ESRD along with urine albumin levels have been shown in meta-analysis of large population^34^. The particular molecular mechanisms and the differences observed in this study are unclear; the diabetes-preconditioned kidney tubular cells could be prone to BSA-induced protein overload-induced tubular damage. The diabetic mice exhibited a significant induction of TGF-βR1 and suppression of miR29s. BSA-induced tubular damage has shown to be associated with the induction of TGF-β1^35^. Therefore, the higher levels of TGF-βRs in the diabetic kidney are preconditioned for TGF-β1-induced fibrogenic signaling and the subsequent stimulation of smad3 phosphorylation. miR29 has been shown to play renoprotective roles, and we have shown that miR29 plays central protective roles in EndMT and suppression of DPP-4 in endothelial cells. In addition, miR29 targets diverse fibrogenic and proinflammatory genes^36^. Therefore, the alterations in the gene expression profiling of diabetic kidney may be preconditioned, accelerating the fibrogenic/EMT programs and subsequent parenchymal damage in our model.

In this study, we focused on the EMT program. EMT has been considered the source of myofibroblasts in the kidney fibrosis process^6^. The contribution of EMT and the presence of tubular-derived fibroblasts remain controversial. However, the EMT program in tubular cells is clearly associated with the production of extracellular matrix proteins. According to Grande *et al*. and Lovisa *et al*., the EMT program, even a partial program, could induce kidney parenchymal damage and kidney fibrosis^10,37^. In tubular cells, the EMT inducer snail plays an important role in tubular damage, tubular cell cycle arrest and damage in neighboring cells^10,38^. In our model, the inhibition of DPP-4 by TENE successfully inhibited kidney fibrosis and EMT associated with the suppression of the EMT inducer (snail, slug, twist, and zeb) and the restoration of epithelial markers (E-cadherin and AQP1) and anti-EMT miRs such as miR-34 and miR-200. Therefore, despite the controversy regarding the contribution of EMT to kidney fibrosis, the alteration of the EMT program by TENE observed in our study is relevant for TENE-mediated kidney protection.

**Figure 7:**
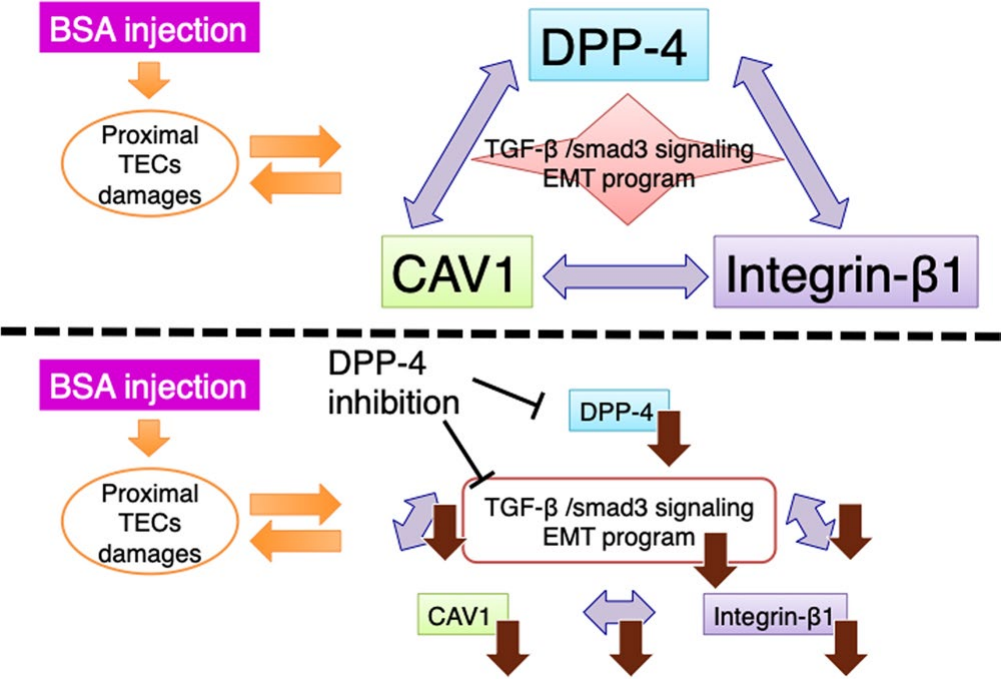
Schematic diagram of the crosstalk among DPP-4, CAV1 and integrin β1 during EMT and fibrosis induced by BSA injection.

In our analysis, the DPP-4 inhibitor blocked the crosstalk among DPP-4, CAV1, and integrin β1, suggesting that DPP-4 could be a key adaptor molecule in the interaction among these three molecules. *In vitro* experiment revealed DPP-4 overexpression and TGF-β1 stimulation induced EMT program and inhibition of DPP-4 or Smad3 phosphorylation suppressed EMT program. Furthermore, by utilizing the Duolink *In Situ* Proximity Ligation Assay, we found close proximity of DPP-4, CAV1 and integrin β1 were increased by DPP-4 overexpression and TGF-β1 stimulation; such proximity were suppressed by inhibition of either DPP-4 or smad3 phosphorylation. Immunoprecipitation analysis also showed that TGF-β1 incubation increased physical interactions among these three molecules and TGF-β neutralization suppressed such interactions induced by DPP-4 overexpression. These results indicate that crosstalk among DPP-4, CAV1 and integrin β1 plays a key role in DPP-4 and TGF-β1-induced epithelial cell signal transduction and in the induction of EMT (Fig. 7). The *in vivo* DPP-4 inhibition also blocked EMT and the associated alteration in fibrogenic molecules and kidney fibrogenic signals in the BSA-injected diabetic mice. Interestingly and unexpectedly, TENE significantly increased the snai1 and snai2 levels without inducing the TGF-β/smad3 signaling pathway in the BSA-injected non-diabetic mice. Regard with this, Long *et al*. reported that DPP-4 inhibitor improve diabetic wound healing via induction of EMT program in the skin wound edge^39^. Wang *et al*. reported that the inhibition of DPP-4 increased tumor metastasis associated with the expression of mesenchymal molecules^40^. Our recent study also demonstrated that DPP-4 inhibitor could induce EMT in breast cancer cells^41^. Therefore, DPP-4 inhibition associated anti-EMT and anti-fibrotic effects could be dependent on the model and disease conditions.

BSA injection is a well-known model in nephritis and chronic serum sickness via the immune system (BSA nephritis)^42^. Although proximal tubular damage can be observed in this animal protocol, we cannot distinguish whether these damages were caused by an albumin-overload or BSA nephritis, which is a limitation of this study protocol. However, the TENE treatment ameliorated renal fibrosis in the BSA-injected diabetic mice without affecting either urine murine albumin or BSA levels. Therefore, at least in this short interval experiment, the TENE-mediated kidney protection was independent of the alteration in the urine albumins (both murine and bovine) filtered from the glomerulus.

In conclusion, we identified a novel molecular mechanism of the renoprotective effect of the DPP-4 inhibitor in BSA-injected diabetic mice in which the crosstalk among DPP-4, integrin β1, CAV1 and the EMT program were inhibited. Therefore, the DPP-4 inhibitor could be a relevant drug for the treatment of diabetic patients with proteinuria.

## Materials and Methods

### Study approval

All animal experiments were approved by the Committee for Animal Experiments of Kanazawa Medical University, and performed in accordance with the Guidelines for Animal Experimentation of Kanazawa Medical University (protocol number 2016–62 and 2017–71). Authors confirm that all the experiments are performed in accordance to Japanese guidelines and regulations for scientific and ethical experimentation.

### Regents and antibodies

For the immunohistochemical and immunofluorescence microscopy analyses a goat polyclonal anti-mouse DPP-4 antibody (AF965, 1:100) was purchased from R&D Systems (Minneapolis, MN, USA). A rabbit polyclonal anti-AQP1 antibody (ab15080, 1:100), a goat polyclonal anti–Snail antibody (ab53519, 1:100), a rabbit polyclonal anti-αSMA antibody, a rabbit polyclonal anti-integrin β1 antibody (ab115146, 1:200) and a rabbit polyclonal anti-CAV1 antibody (ab2910, 1:200) were purchased from Abcam (Cambridge, MA, USA). A rat polyclonal anti-E-cadherin antibody (GTX11512, 1:100) was purchased from GeneTex (Irvine, CA, 1:100). For the western blotting and Duolink proximity ligation assay, a goat polyclonal anti-DPP-4 antibody (SAB2500328, 1:500) was purchased from Sigma-Aldrich (St. Louis, MO, USA). A goat polyclonal anti-CAV1 antibody (ab36152, 1:500) was purchased from Abcam (Cambridge, MA, USA). A rabbit smad3 antibody (#9513, 1:1000) and a β-actin rabbit mAb (HRP conjugate) (#5125, 1:3000) were purchased from Cell Signaling Technology (Danvers, MA). A rabbit polyclonal anti-phospho smad3 (s423 and s425, 1:500) antibody was purchased from Rockland Immunochemicals (Gilbertsville, PA). For the multiplex immunohistochemistry staining, an opal polymer HRP Ms + Rb, an opal 520 reagent, an opal 570 reagent, an opal 690 reagent and a spectral DAPI solution were purchased from PerkinElmer (Waltham, MA, USA). For the immunoprecipitation, a rabbit polyclonal anti-CAV1 antibody (ab2910, 1:100) was purchased from Abcam (Cambridge, MA, USA) and A rabbit monoclonal anti-DPP-4 antibody (#67138, 1:100) was purchased from Cell Signaling Technology (Danvers, MA). Recombinant human TGF-β1 (#100-21) was purchased from PeproTech (Rocky Hill, NJ, USA). Smad3 inhibitor SIS3 (CAS 1009104-85-1) was purchased from Santa Cruz Biotechnology (Dallas, TX, USA).

### Animal experiment

Eight-week-old male CD-1 mice (Sankyo Laboratory Service, Tokyo, Japan) were used in all *in vivo* experiments. The mice were injected with STZ [200 mg/kg BW] intraperitoneally. The diagnosis of diabetes was confirmed by a blood glucose level >16 mmol/L 2 weeks after the STZ injection. At 4 weeks after the induction of diabetes, the diabetic mice were divided into two groups (TENE [30 mg/kg BW/day in drinking water] and untreated). TENE was diluted directly in the drinking water. Simultaneously, the mice received an FFA-bound BSA injection (0.3 g/30 g BW). BSA was purchased from Sigma-Aldrich (St. Louis, MO, USA). BSA was injected intraperitoneally for 11 days of 14 days, and subsequently, we observed tubular damage associated with inflammation, apoptosis and fibrosis.

All mice were sacrificed 2 weeks after the BSA injection and initiation of the TENE (Mitsubishi Tanabe Pharma, Tokyo, Japan) treatment. Before sacrificing the mice, their blood pressure was monitored via the tail cuff method using BP-98A (Softron Co., Beijing, China). The blood glucose levels were measured using a portable glucose meter (Antisense III, HORIBA, Ltd., Kyoto, Japan). All samples were collected and stored at −80 °C until use.

### Measurement of urine albumin to creatinine ratio

The murine specific urinary albumin level was measured using a murine microalbuminuria ELISA kit (albuwell M Test kit, Exocell, Inc. Philadelphia; Cosmo Bio Co., LTD). The urinary creatinine levels were measured using a QuantiChromTM Creatinine Assay Kit (BioAssay System). We used SoftMax pro 6.4 to analyze the urinary albumin and creatinine levels.

### Measurement of urine BSA level

The urine BSA levels were measured using a specific ELISA kit for BSA (Arigobio, Hsinchu City, Taiwan). We used SoftMax pro 6.4 to analyze the urinary albumin and creatinine levels.

### Measurement of serum AST, ALT and NT-proBNP level

The serum AST, ALT and NT-proBNP were measured using a specific ELISA kit for AST, ALT and NT-proBNP (MyBioSource, CA, USA).

### Histopathology

The kidney, Liver and heart were fixed in 10% formaldehyde and embedded in paraffin. For the Masson’s trichrome staining MTS, SR staining and immunohistochemistry, all tissues were cut into 5 μm thick sections. The SR staining was performed using a Picrosirius Red Stain Kit (Philadelphia; Cosmo Bio Co., LTD). Six MTS or SR stained 200× visual areas from each mouse were analyzed to calculate the fibrotic area using ImageJ software. The tubular atrophy scores were calculated according to the chronic allograft damage index in 200× visual fields.

### Immunohistochemistry and multiplex staining

Formaldehyde-fixed, paraffin-embedded (FFPE) kidney sections (5 μm thick) were deparaffinized and rehydrated (2 min in xylene, four times; 1 min in 100% ethanol, twice; 30 s in 95% ethanol; 45 s in 70% ethanol; and 1 min in distilled water), and the antigen was retrieved in a 10 mM citrate buffer pH 6 at 98 °C for 60 min. To block the endogenous peroxidase, all sections were incubated in 0.3% hydrogen peroxide for 10 min. The immunohistochemistry was performed using a Vectastain ABC Kit (Vector Laboratories, Burlingame, CA). The primary antibody was diluted as mentioned above. In the negative controls, the primary antibody was omitted and replaced with the blocking solution. For the multiplex staining, an opal *in situ* kit was purchased from PerkinElmer (Waltham, MA, USA). FFPE slides were deparaffinized, and the antigen was retrieved as described above. αSMA and DPP-4 were labeled with opal 520 (TSA-FITC), E-cadherin and integrin β1 were labeled with opal 570 (TSA-Cy3), CAV1 was labeled with opal 670 (TSA-Cy5), and the nuclei were labeled with DAPI.

### Overexpression experiment

CAV1 DNA plasmid was purchased from Origene.The pCMV6-DPP-4-GFP plasmid was purchased from ORIGENE (Rockville, MD). To generate the pCMV-Myc-DPP-4 plasmid, we amplified the full-length DPP-4 cDNA by PCR using the pCMV6-DPP-4-GFP plasmid as a template and the specific primer pair (Fw. 5′- C CGA ATT CGG ATG AAG ACA CCG TGG AAG GTT CTT C -3′; Rev 5′- AT CTC GAG CTA AGG TAA AGA GAA ACA GTT TTT TAT G -3′). Both the amplified DPP-4 cDNA and pCMV-Myc cloning vector (Clontech, Mountain View, CA) were digested with EcoRI and XhoI, and the resulting product was ligated (TOYOBO, Japan). The ligated products (pCMV-Myc-DPP-4) were transformed into competent cells and amplified, and the sequence was confirmed.

### Proximity ligation assay

Duolink *In Situ* kits were used to detect the proximity of DPP-4/integrin β1, DPP-4/CAV1 and CAV1/integrin β1 *in vivo* following the manufacturer’s protocol^14^. Briefly, cells from the human renal tubular epithelial cell line (HK-2) (CRL-2190TM; ATCC) were cultured in Keratinocyte Serum-Free Medium (K-SFM; Invitrogen; Cat# 17005-042) supplemented with 0.05 mg/ml of bovine pituitary extract (BPE) and 5 ng/ml human recombinant epidermal growth factor (EGF). When the HK-2 cells on the 8-well Culture Slides (BD Falcon, New York, USA) reached 70% confluence, 10 ng/mL recombinant human TGF-β1 was added to the experimental medium for 48 h (HuMedia-MVG in serum-free RPMI at a 1:3 ratio) with or without a TENE (0.1 μM) preincubation for 2 h. Vehicle (PBS) was added to the control well. The cells were washed with PBS, fixed with 4% paraformaldehyde, permeabilized with 0.2% Triton-X100 and blocked with the blocking solution from the Duolink *In Situ* Kit. Subsequently, the cells were incubated with the primary antibodies (i.e., goat polyclonal anti-DPP-4 antibody/rabbit polyclonal anti-integrin β1 antibody, goat polyclonal anti-DPP-4 antibody/rabbit polyclonal anti-CAV1 antibody or goat polyclonal anti-CAV1 antibody/rabbit polyclonal anti-integrin β1 antibody) at 4 °C overnight. The slides were mounted with DAPI and analyzed by fluorescence microscopy. For each slide, the original magnification of ×400 pictures was obtained from 6 different areas, and quantification was performed.

### Immunoprecipitation

The immunoprecipitation assay was performed as previously described^16^. HK-2 cell were treated with TGF-β (10 ng/ML) and DPP-4 overexpression vector. At 48 h post-treatment or transfection, cells were rinsed with ice cold PBS and lysated with ice-cold cell lysis buffer and then collected. The samples were sonicated on ice three times for 5 s and microcentrifuged for 10 min at 14,000 g. We used 500 μL of the supernatant for immunoprecipitation and incubated with goat- anti DPP-4 antibody overnight. Then Protein A was added and incubated for 1–3 h at 4 °C and then microcentrifuged for 30 s at 4 °C. The pellet was then washed and resuspended with SDS sample buffer, and sample was then analyzed by western blotting.

### Microarray analysis

For the microarray analysis, total RNA was extracted from the mouse kidneys (n = 2) using RNAlater-ICE (Invitrogen) and the RNeasy Lipid Tissue Mini Kit (Qiagen, Hilden, Germany) according to the manufacturer’s instructions. An Agilent 2100 Bioanalyzer was used to evaluate the quality of the obtained RNA. The RNA concentration was measured using a NanoDrop 1000 Spectrophotometer. A GeneChip analysis was performed using a GeneChip WT PLUS Reagent Kit, GeneChip analysis Mouse Gene 2.0 ST Array and GeneChip Hybridization, Wash, and Stain Kit (Affymetrix, California, USA). The images were acquired and quantified using a GeneChip Scanner 3000 7 G and GeneChip Command Console. The statistical data mining and analysis were processed by GeneSpring GX Version12.6 (Agilent, California, USA) and David. A heat map was generated by GraphPad Prism7.

### RNA and miR isolation and Quantitative PCR (qPCR)

RNA was extracted from frozen kidneys using TRIzol (Life Technologies, 15596-018, Waltham, MA) according to the manufacturer’s instructions. The RNA concentration was quantified using a NanoDrop 1000 Spectrophotometer. cDNA was generated using a PrimeScript RT Reagent Kit (TAKARA, RR037A, Shiga, Japan). The gene expression was quantified using a SYBR Green PCR kit using 10 ng of cDNA. The primers used for the quantification were designed by Hokkaido System Science Co., Ltd. (Sapporo, Japan). All experiments were performed in duplicate, and 18S ribosomal RNA (Qiagen) was utilized as an internal control. MiR was extracted using a miRNeasy Mini kit (Qiagen) according to the manufacturer’s instructions. The cDNA was generated using a miScript II RT kit (Qiagen). The miScript SYBR Green PCR Kit (Qiagen) was used to quantify the miR expression using 3 ng of cDNA. The primers used to quantify Mm_miR-200b-1, Mm_miR-200b-3, Mm_miR-29-a, Mm_miR-29-b, Mm_miR-29-c, Mm_miR-34-a, Mm_miR-34-b and Mm_miR-34-c were included in the miScript primer assays (Qiagen). All experiments were performed in duplicate, and Hs_RNU6-2_1 (Qiagen) was utilized as an internal control (Supplementary Table).

### Statistical analysis

All data are expressed as the mean ± SEM. Prism 7 software was used for the statistical analysis. The differences among the groups were analyzed by performing one-way analysis of variance (ANOVA) followed by Tukey HSD testing for multiple comparisons unless otherwise indicated in the legend. Comparisons with P-values < 0.05 were considered statistically significant.

### Duality of interest

This paper is collaborated with Mitsubishi Tanabe Pharma Corporation

## Supplementary information

Supplementary Figure

## Data Availability

Authors declare that all data is available.
