# Supplementary material for: Dipeptidyl peptidase-4 plays a pathogenic role in BSA-induced kidney injury in diabetic mice
Source: Sci Rep. 2019 May 17;9:7519. doi: 10.1038/s41598-019-43730-5 (PMC6525172; doi:10.1038/s41598-019-43730-5)

**Supplementary Information for:**

**Dipeptidyl peptidase-4 plays a pathogenic role in BSA-induced kidney injury in diabetic mice**

Yuta Takagaki^1^, Sen Shi^1^, Makoto Katoh^3^, Munehiro Kitada^1,2^, Keizo Kanasaki*^1,2^, and Daisuke Koya*^1,2^

1. *Department of Diabetology and Endocrinology & 2) Division of Anticipatory Molecular Food Science and Technology, Kanazawa Medical University, Uchinada, Ishikawa, Japan 920-0293*

*3)*  *Mitsubishi Tanabe Pharma Corporation Ikuyaku. Integrated Value Development Division*

*Correspondence:

Keizo Kanasaki, M.D., Ph.D. E-mail: [kkanasak@kanazawa-med.ac.jp](mailto:kkanasak@kanazawa-med.ac.jp)

or

Daisuke Koya, M.D., Ph.D. E-mail: [koya0516@kanazawa-med.ac.jp](mailto:koya0516@kanazawa-med.ac.jp)

Department of Diabetology & Endocrinology, Kanazawa Medical University, Uchinada, Ishikawa 920-0293, Japan; TEL.: 81-76-286-2211 (Ext. 3305); FAX: 81-76-286-6927

**
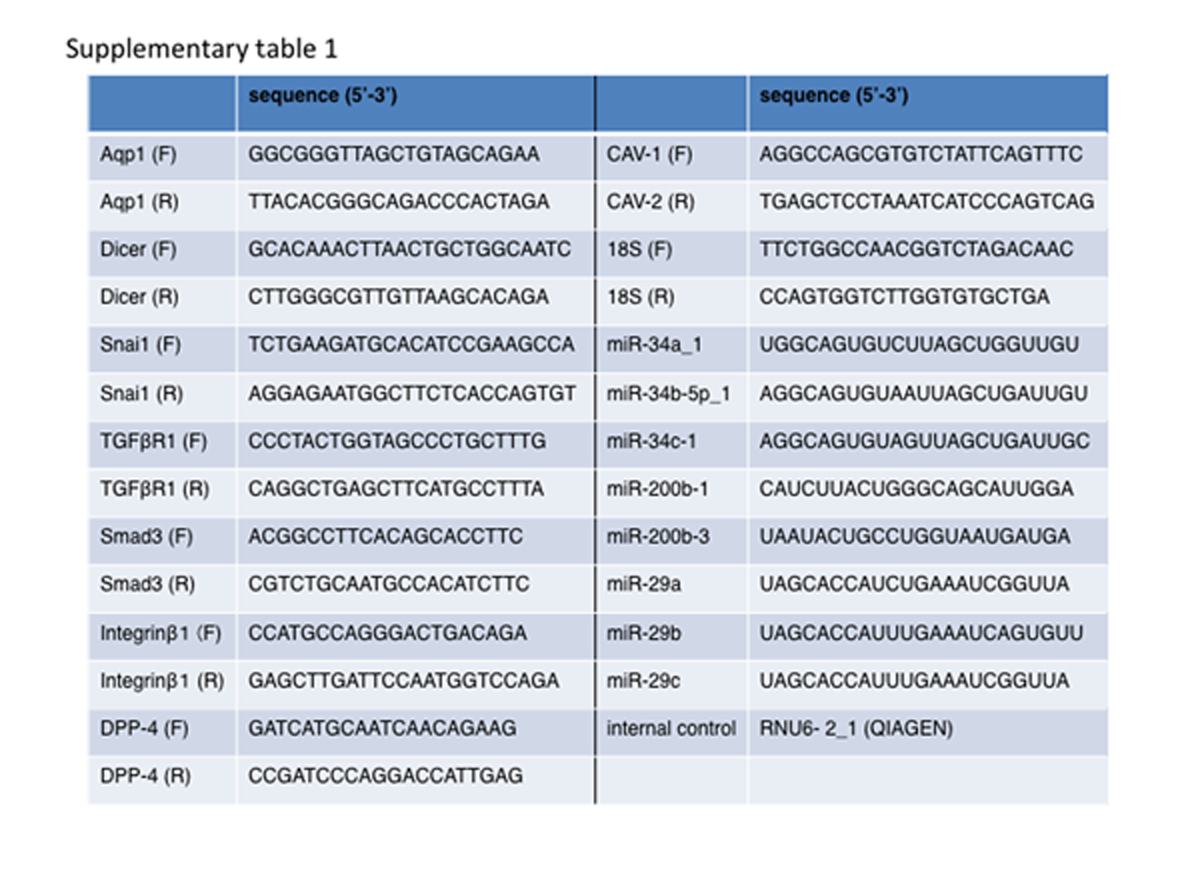
**

**Supplementary Table. List of primer sequences**

**
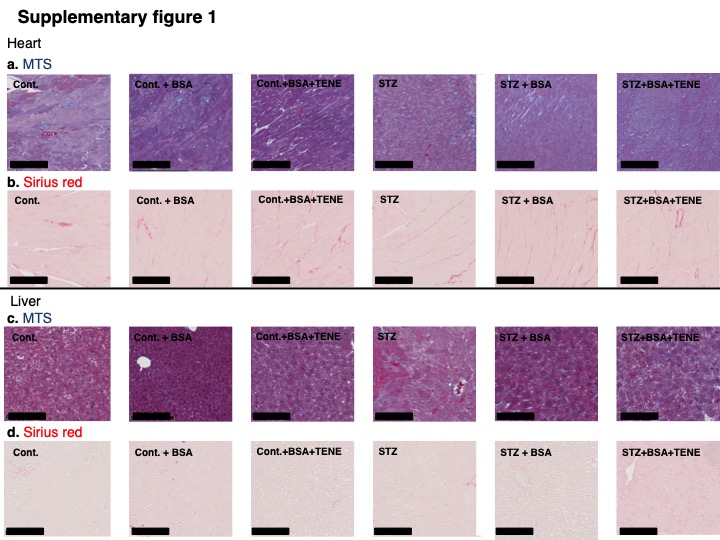
**

**Supplementary figure Fig 1. Diabetes and/or BSA injection influence no significant alteration in the histology of heart and liver in mice.**

**(a, b)** Representative images of heart of each group. Scale bars, 250μm. **(c, d)** Representative images of each group. Scale bars, 100μm. *n*=7 mice per each group

**
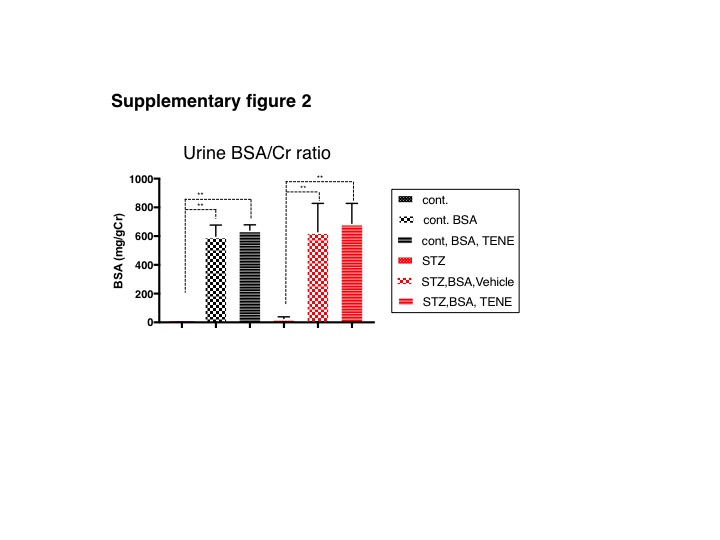
**

**Supplementary figure Fig 2. Urine BSA ELISA**

Urine BSA levels were measured in the BSA-injected or untreated control and diabetic mice with or without the TENE treatment, *n =* 4 or 5. Urine BSA levels were divided according to the urine creatinine levels (urine BSA/creatinine ratio). Urine BSA/creatinine ratios were not significantly changed among the BSA-injected mice. The TENE treatment did not affect the urine excretion levels of BSA.


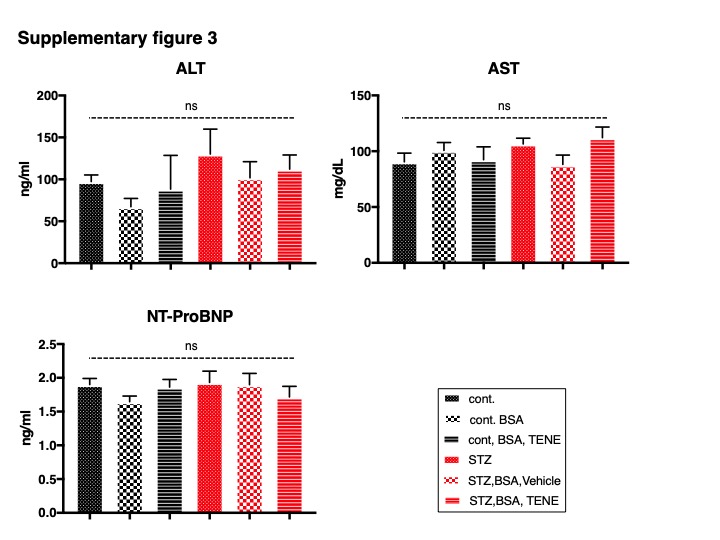


**Supplementary figure Fig 3. There was no significant difference of liver injury markers, AST and ALT, and heart failure marker, NT-ProBNP, among all groups.** **(a-c)** Plasma ALT, AST, NT-ProBNP levels were measured in the BSA-injected or untreated control and diabetic mice with or without the TENE treatment. Plasma ALT, AST and NT-ProBNP were not significantly changed among all groups. *n*=7 mice per each group


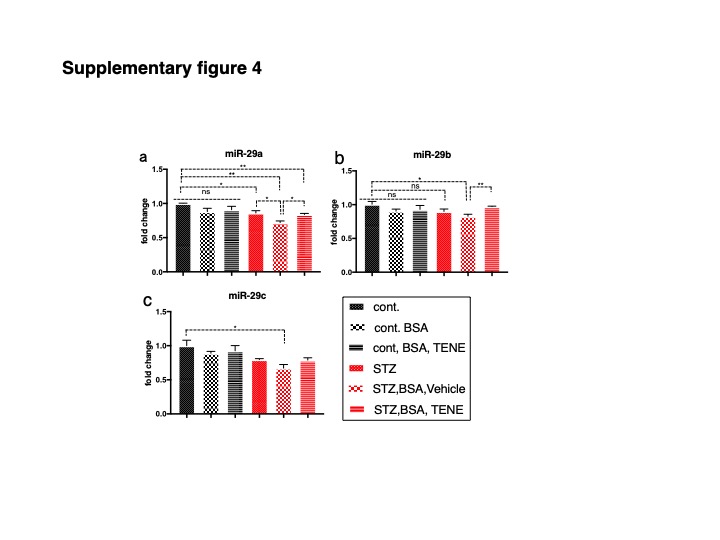


**Supplementary figure Fig 4. BSA injection decreased miR-29s levels in diabetic mice; TENE restored these changes**

**(a-c)** qPCR analysis of the expression of the indicated genes in the kidney of mice in each group (*n* = 7 mice per group). Gene expression was normalized to the control mice value.


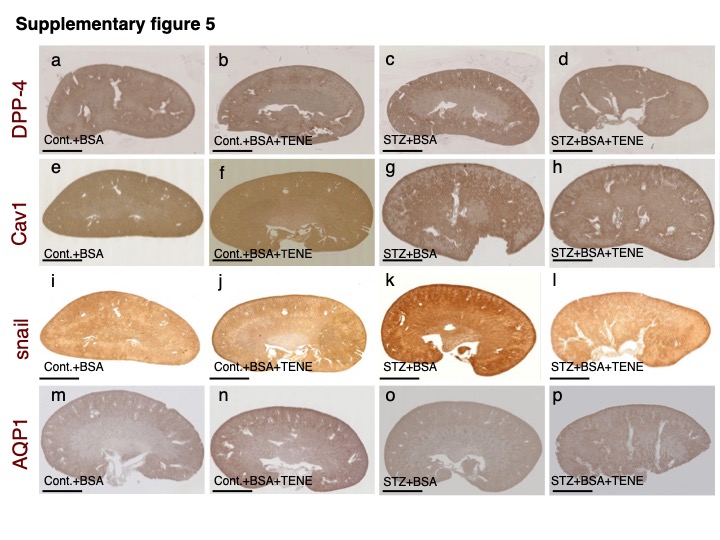


**Supplementary figure Fig 5. Images of entire kidney of immunohistochemistry analysis.**

Representative images of each group. Scale bars, 2.5 mm.


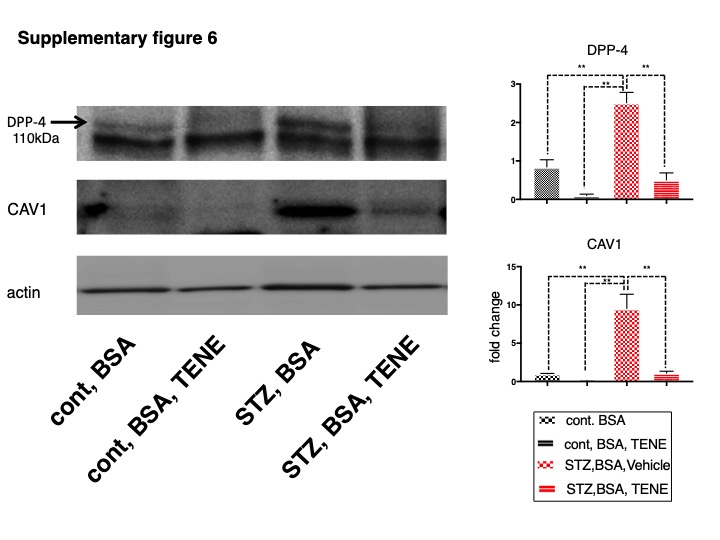


**Supplementary figure Fig 6. BSA-injected diabetic mice exhibited high protein expression levels of DPP-4 and CAV1.**

Western blot analysis of mice kidney (n=7).


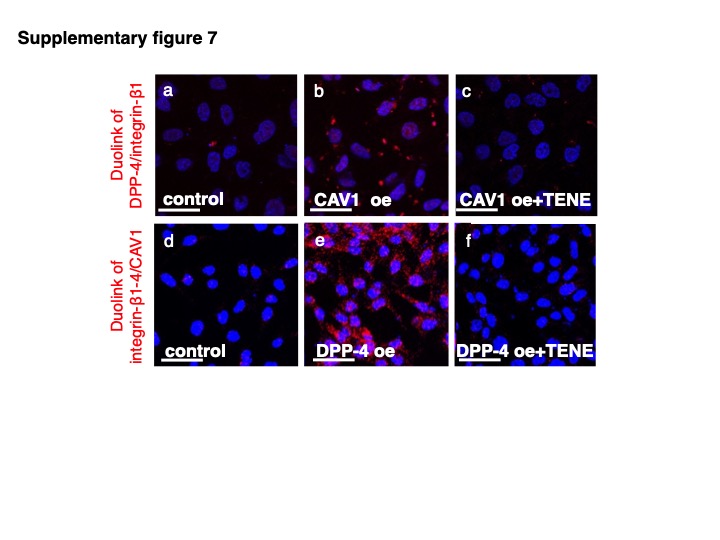


**Supplementary figure Fig 7. TENE treatment suppressed the crosstalk among DPP-4, integrin β1 and CAV1 induced by CAV1 and DPP-4 overexpression *in vitro*.** Duolink in situ analysis of **(a-c)** DPP-4/integrin β1, **(d-f)** integrin β1/CAV1 and in HK-2 cells with or without CAV1 (a-c) and DPP-4 (d-f) overexpression was performed by confocal microscopy (×1260). Scale bar: 50 μm in each panel.


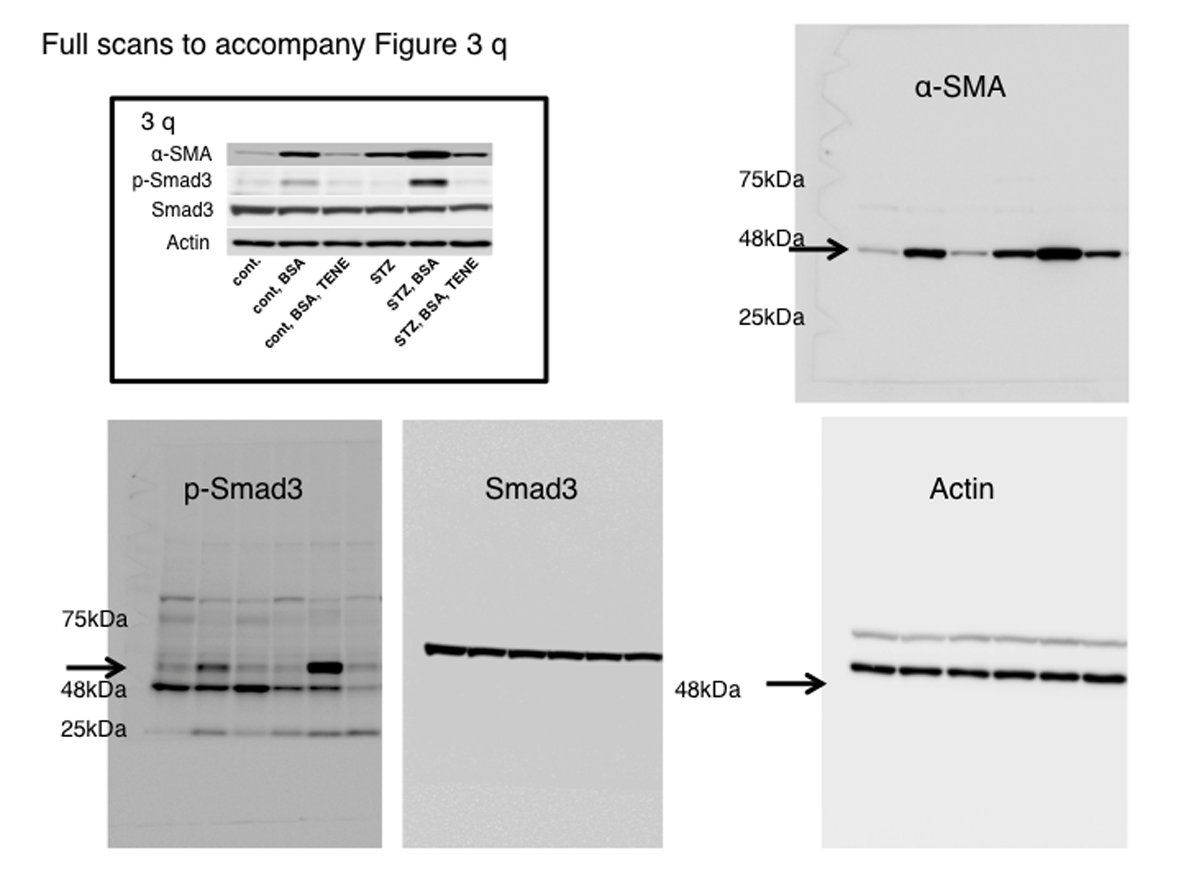


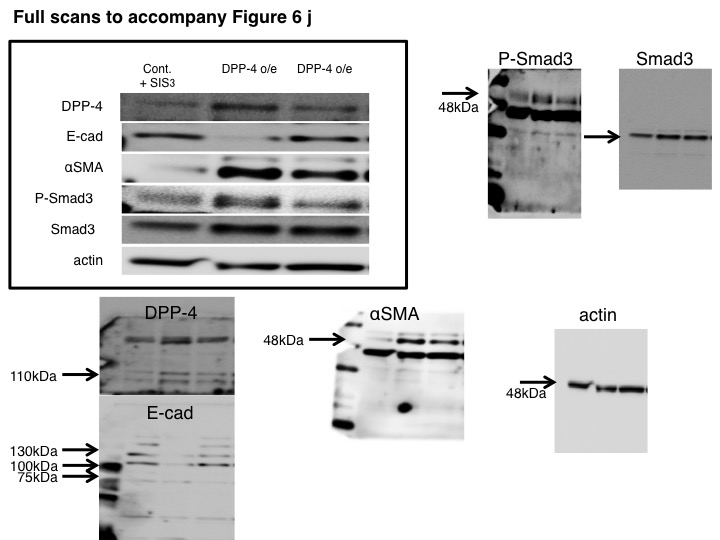


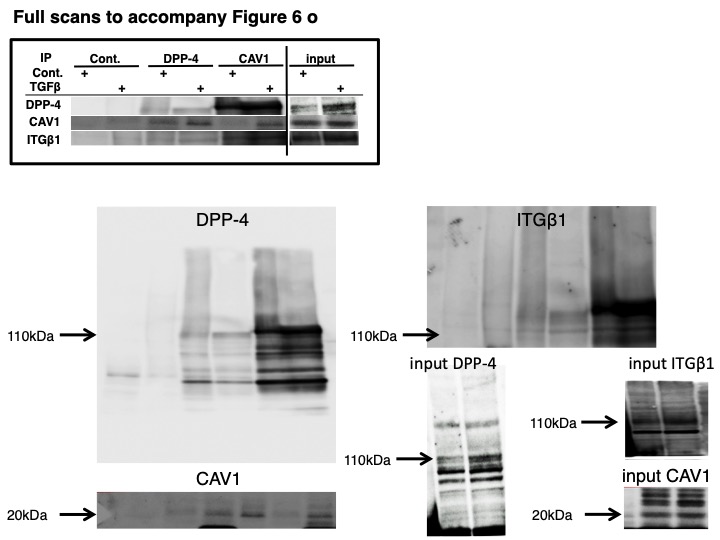


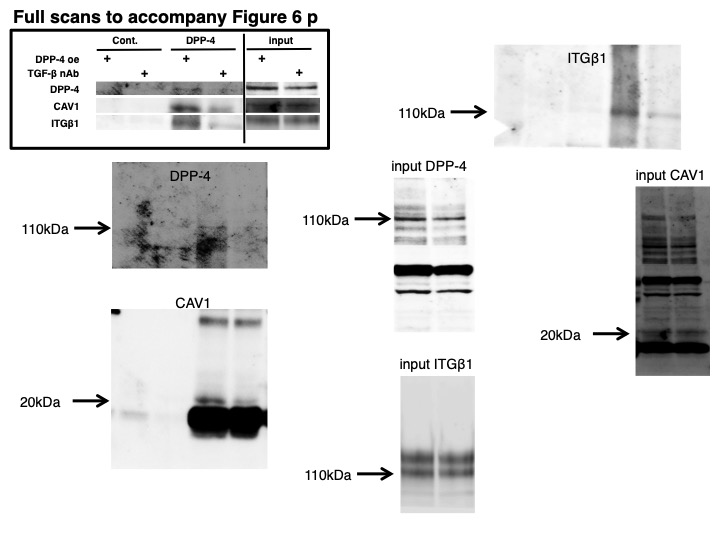

Supplement: Supplementary file 1 — Supplementary Figure [file 41598_2019_43730_MOESM1_ESM.docx]
